# Peptide receptor radionuclide therapy (PRRT) in radioiodine-refractory thyroid cancer: A case report of significant response to lu177 DOTA-TATE treatment

**DOI:** 10.20945/2359-3997000000520

**Published:** 2022-09-08

DOI: 10.20945/2359-3997000000451

Arch Endocrinol Metab. 2022;66(2):269-71

Where you read:

**Mohammad Esmatinia** ^1^

https://orcid.org/0000-0001-8582-5658

Should read:

**Mahdi Mottaghi** ^1^

https://orcid.org/0000-0001-8582-5658

